# A semiquantitative color Doppler ultrasound scoring system for evaluation of synovitis in joints of patients with blood-induced arthropathy

**DOI:** 10.1186/s13244-021-01043-0

**Published:** 2021-09-25

**Authors:** Ningning Zhang, Sheng Yang, Anne-Fleur Zwagemaker, Aihua Huo, Ying-Jia Li, Fang Zhou, Pamela Hilliard, Sandra Squire, Vanessa Bouskill, Arun Mohanta, Alex Zhou, Jose Jarrin, Runhui Wu, Jing Sun, Brian Luke, Rahim Moineddin, Victor S. Blanchette, Yun Peng, Andrea S. Doria

**Affiliations:** 1 Department of Radiology, Beijing Children’s Hospital, Capital Medical University, National Center for Children’s Health, Beijing, China; 2 Department of Ultrasound, Chengdu Women’s and Children’s Central Hospital, Sichuan, China; 3 Department of Pediatric Haematology, Amsterdam UMC, University of Amsterdam, Amsterdam, Netherlands; 4 Department of Radiology, Nanfang Hospital, Guangzhou, China; 5 Department of Rehabilitation, The Hospital for Sick Children, Toronto, ON Canada; 6 Service of Physiotherapy, St Paul’s Hospital, Providence Health Care, Vancouver, BC Canada; 7 Department of Paediatrics, University of Toronto, Toronto, ON Canada; 8 Division of Haematology/Oncology, The Hospital for Sick Children, Toronto, ON Canada; 9 Department of Diagnostic Imaging, Research Institute, The Hospital for Sick Children, University of Toronto, 555 University Ave, Toronto, ON M5G 1X8 Canada; 10 Haematology/Oncology Center, Beijing Children’s Hospital, Capital Medical University, National Center for Children’s Health, Beijing, China; 11 Division of Haematology and Transfusion Medicine, Children’s Hospital of Eastern Ontario, Ottawa, ON Canada; 12 Division of Family and Community Medicine, University of Toronto, Toronto, ON Canada; 13 Department of Medical Imaging, University of Toronto, Toronto, ON Canada

**Keywords:** Color Doppler ultrasound, Synovial hypertrophy, Magnetic resonance imaging (MRI), Hemophilic arthropathy, Children and adolescents.

## Abstract

**Background:**

Intra-articular bleeds in patients with inherited bleeding disorders lead to active synovitis which may progress to a chronic state over time. We explored the diagnostic value of color Doppler ultrasound in detecting synovitis in boys with bleeding disorders.

**Results:**

Sixty boys with hemophilia and 3 boys with type 3 von Willebrand disease aged 5 to 18 years (median 12.3 years) were imaged by gray-scale and color Doppler ultrasound (US) in three centers (Beijing, China [*n* = 22], Guangzhou, China [*n* = 12] and Toronto, Canada [*n* = 29])) in this observational study. Images were independently reviewed by two radiologists blinded to clinical data using a subjective semi-quantitative scoring system and objective measurements of synovial thickness and vascularity. Inter-reader reliability for using subjective versus objective color Doppler US methods for assessing synovial vascularity was excellent for the subjective method and moderate/lower range of substantial for the objective method. Agreement between degree of vascularity on color Doppler and extent of synovial hypertrophy on gray-scale US was overall poor for Canada data and moderate for China data. Correlations between degree of vascularity on color Doppler and synovial hypertrophy on gray-scale US, and clinical constructs (total and itemized HJHS scores and total Pettersson X-ray scores) for assessment of blood-induced arthropathy were all poor.

**Conclusion:**

Color Doppler US is a valuable scoring method for evaluating reactive synovitis in joints of subjects with inherited bleeding disorders and holds potential for assessing post-bleed reactive synovitis once further information on its association with timing of the joint bleed becomes available in the literature.

**Supplementary Information:**

The online version contains supplementary material available at 10.1186/s13244-021-01043-0.

## Key points

- Active synovitis due to intra-articular bleeds may progress to a chronic state.
- Different correlation levels were obtained while comparing subjective versus objective assessing methods.
- Color Doppler US holds potential for assessing post-bleed reactive synovitis.

## Background

Recurrent joint bleeding and subsequent development of joint damage are complications in persons with moderate or severe hemophilia, in whom hemarthroses account for 70–80% of all bleeding episodes [1]. Intra-articular bleeds lead initially to active synovitis, characterized by hypervascularization and inflammatory activity, and microscopic degenerative changes in the cartilage. Over time, there may be progression to a chronic state of synovitis, where macroscopic cartilage erosions and arthropathy may be observed [2, 3]. Due to the occurrence of subclinical bleeding, early pathological synovial changes may go unrecognized. Nevertheless, such subtle undiagnosed synovial abnormalities may lead to irreversible arthropathy later on [4]. Hence, accurate monitoring of joint status is an essential part of hemophilia care in order to guide treatment decision-making.

Although contrast-enhanced magnetic resonance imaging (MRI) is a sensitive imaging technique to assess early synovial joint disease in hemophilia [5], its applicability is restricted by high costs for data scanning and interpretation, and the need for sedation in young children, and for administration of contrast for assessment of synovitis. Technologic advances in real-time sonography have facilitated the use of gray-scale sonography and color Doppler for evaluating the musculoskeletal system [6–8]. Ultrasound with color Doppler can be a useful non-invasive technique in this regard. Acharya et al. have shown that power Doppler ultrasound is an inexpensive imaging tool for detecting hemophilic synovitis and for quantifying synovial vascularity in knees, elbows and ankles of persons with hemophilia [9]. Color and power Doppler ultrasound hold potential for detection and quantitation of increased vascularity in joints of persons with hemophilia related to synovitis which can be reactive to recent intraarticular bleeds. This has been previously shown in animal [10, 11] and human [12] studies. Power Doppler has improved sensitivity compared with conventional Doppler frequency displays [13], however it shows a high degree of frame-averaging requiring that the patient keeps still during the performance of the examination, a task that can be challenging for young children. The contrast resolution and sensitivity of modern color flow systems have rapidly improved over the last decade, improving the range and quality of vascular examination [14]. Torp-Pedersen et al. assessed 6 different types of high- and intermediate-range ultrasound machines to determine how settings for power and color Doppler ultrasound sensitivity varied in patients with rheumatoid arthritis affecting the wrist joint. Their study results showed that whereas power Doppler was more sensitive on half of the machines color Doppler was more sensitive on the other half, using both manufacturer and local study settings [15]. Because color Doppler is substantially less affected by motion artifacts than power Doppler it is preferentially used in the assessment of pediatric patients in some centers.

Although rheumatoid arthritis and juvenile idiopathic arthritis (JIA) are characterized by prominent synovitis and hemophilic arthritis by minor synovitis, the joint disease in these entities shows similarities in both radiographic and histological appearance [16, 17]. In JIA and adult rheumatoid arthritis, color pixel intensity obtained with color and power Doppler ultrasound was found to be markedly increased in patients with active disease and patients having quiescent disease with serum markers of active disease [18, 19]. In blood-induced arthritis, animal studies have shown that ongoing or repeat joint bleed induces synovial changes that include inflammation, hyperplasia and angiogenesis [20–22]. Acharya et al. noted local and systemic angiogenic response in hemophilic subjects with recurrent hemarthroses points towards mechanisms of onset and progression of hemophilic synovitis [23]. As neoangiogenesis and inflammatory processes require increased blood flow [23], the presence of synovial hyperemia on color Doppler ultrasound can be a biomarker of active synovitis raising the hypothesis that Doppler sonography may add value in the assessment of arthropathy in persons with hemophilia.

Concerning the use of scoring systems, previous studies have used color Doppler scoring systems for assessment of small joints in the hands and feet of subjects with rheumatoid arthritis [24]. However, to our knowledge, few if any previous studies have evaluated the clinical value of color Doppler ultrasound in comparison to MRI for assessment of reactive synovitis in index joints (ankles, knees and elbows) in subjects with hemophilia using an a priori devised standardized color Doppler scoring system.

The specific aims of this study were (i) to evaluate the agreement and associations between different color Doppler methods for assessment of the maximum degree of synovial vascularity, a semi-quantitative scoring method (subjective assessment) and quantitative pixel measurements (objective assessment), and (ii) to correlate the degree of vascularity on color Doppler (by semi-quantitative and quantitative methods), and the extent of synovial hypertrophy on gray-scale ultrasound, with clinical/ other imaging constructs (Hemophilia Joint Health Score [HJHS], Pettersson X-ray scores and MRI of the study joint).

## Methods

Research Ethics Board approval for the study was obtained in all participating centers. The approval dates were respectively April 2, 2007 (for the prospective part) and February 5, 2015 (for the current study including research agreements with the centers China) in Toronto, Canada; December 29, 2014 in Beijing, China and December 25, 2014 in Guangzhou, China. Informed consent for participation in the study was obtained for all subjects.

### Patients

Eligibility for this study included patients with bleeding disorders (hemophilia A, hemophilia B and von Willebrand [vWD] disease) with ages ranging between 0 and 18 years. The ultrasound examinations whose data were used in this study were prospectively acquired in Canada and in China. The recruitment period of patients in Toronto, Canada was September 19, 2007 to January 23, 2009; in Beijing, China, February 7 to 12, 2015 and in Guangzhou, China, February 11 to 13, 2015. Afterwards, the data were retrospectively accrued for the purpose of this study, based on an opportunity sample size. This has limited the assessment of timing of intraarticular bleeds through a rigorous methodology that enabled uniform documentation in patients’ clinical records.

### Imaging acquisition

Ultrasound, MRI and physical examination of the study joints were obtained on the same day, and radiographs of the corresponding study joint were obtained within an interval of 3 days of the other diagnostic tests. Images were acquired in Toronto during the period of September 2007 to January 2009 and in China in February 2015.

### Ultrasound

Gray and color Doppler ultrasound images were obtained of ankles, knees and elbows according to protocols previously described [25–27]. The protocol included sagittal, coronal and axial images obtained with depths and gains adjustable to the patients’ biotypes. Sites 1 Beijing Children’s Hospital, Beijing and 2, Nanfang Hospital, Guangzhou, in China used a 12–5-MHz linear-array transducer on an iU22 scanner (Philips Medical Systems, Bothell, WA), and site 3, Hospital for Sick Children, Toronto, Canada used a 17–5 MHz linear-array transducer on an iU22 scanner (Philips Medical Systems, Bothell, WA). Each ultrasound scan was performed by two operators blinded to any clinical or additional imaging information: in the Chinese hemophilia treatment centers (Beijing Children’s Hospital, Beijing and Nanfang Hospital, Guangzhou) where the data were acquired between February 7 and 13, 2015 one operator was from China (in site 1, N.Z. and in site 2, Y-J.L) and one from Canada (in site 1, A.M. and in site 2, J.J. or A.Z.), and in the Canadian hemophilia treatment center (Hospital for Sick Children, Toronto) where the data were acquired between September 19, 2007 and January 23, 2009, the two operators were from Canada (A.M. and J.J.).

The most severely affected joint (knee, elbow or ankle) of the participating patients as per clinical history and prior X-rays, MRI, and ultrasound scans, according to a priori established protocols, were scanned in this study [25–27]. Scanning time was approximately 20 min for ankles and elbows, and 30 min for knees.

### MRI

MRI assessments were obtained on 3.0 Tesla scanners: in site 1 investigators used an Achieva TX (Philips Medical Systems, Bothell, WA) with an 8-channel coil for knees, a flexible coil for elbows and a head coil for ankles. In sites 2 and 3 a Trio Tim system (Siemens, Erlangen, Germany) was employed with a 15-channel coil for knees, a flexible coil for elbows and a head coil for ankles. MRI scanning protocols used in the three hemophilia treatment centers are detailed in the Additional file 1: Table 1.

### Imaging analysis

#### Gray scale ultrasound

##### Quantitative assessment

Synovial thickness was measured on the plane and image that depicted the area with the most severely hypertrophic synovium by a single operator (S.Y.).

#### Color Doppler ultrasound

Synovial vascularity of study joints was measured on the plane that depicted the most marked vascularity both quantitatively and semi-quantitatively by two operators (N.Z., S.Y.).

##### Quantitative assessment

Geometric regions-of-interest (ROIs) encompassing unique color pixel areas for each case were manually drawn within the color boxes to quantify the pixel intensity on color Doppler, including area of pixels (in mm^2^) and the pixel count as provided by the built-in software of our Picture Archive Communications Systems (PACS), General Electric, Milwaukee, WI, U.S.A.

##### Semi-quantitative assessment

**Fig. 1:**
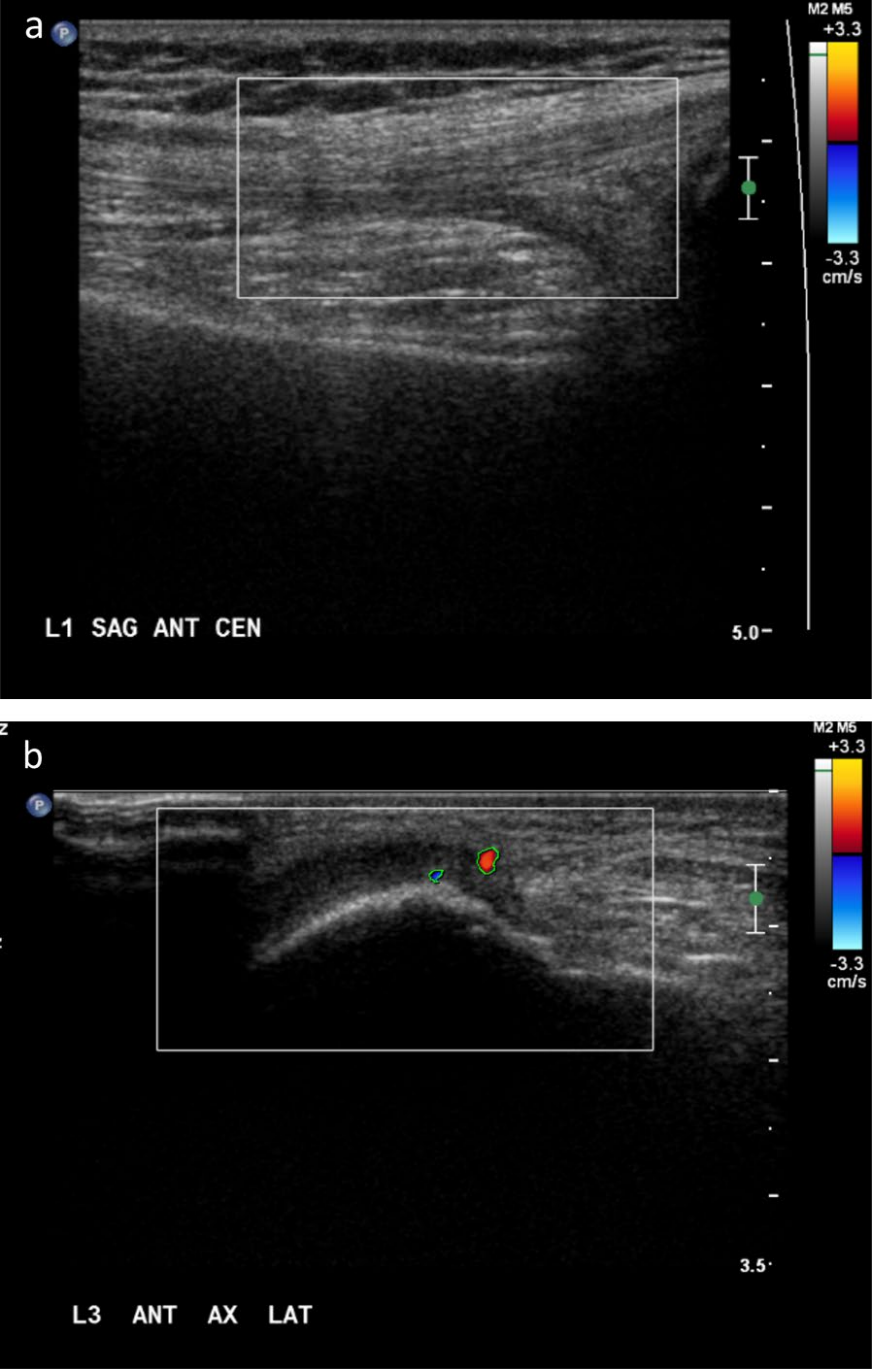
Normal synovial vascularity of joints of patients with hemophilic arthropathy on color Doppler ultrasound. **a** Thirteen-year old boy with severe hemophilia A and a history of recurrent bleeding into his right knee. Color Doppler sonogram obtained on the sagittal anterior central plane at the L1 level shows no color pixel dot within the color box. Score is 0. Objective area of pixels is 0, score equals 0. **b** Fourteen-year old boy with severe hemophilia A and a history of recurrent bleeding into his right knee. Color Doppler sonogram obtained on the axial anterior-lateral plane at the L3 level shows 2  color pixel dots (less than 4 dots) within the color box, Score equals 0. The area of color pixels within the color box is 569 units^2^

**Fig. 2:**
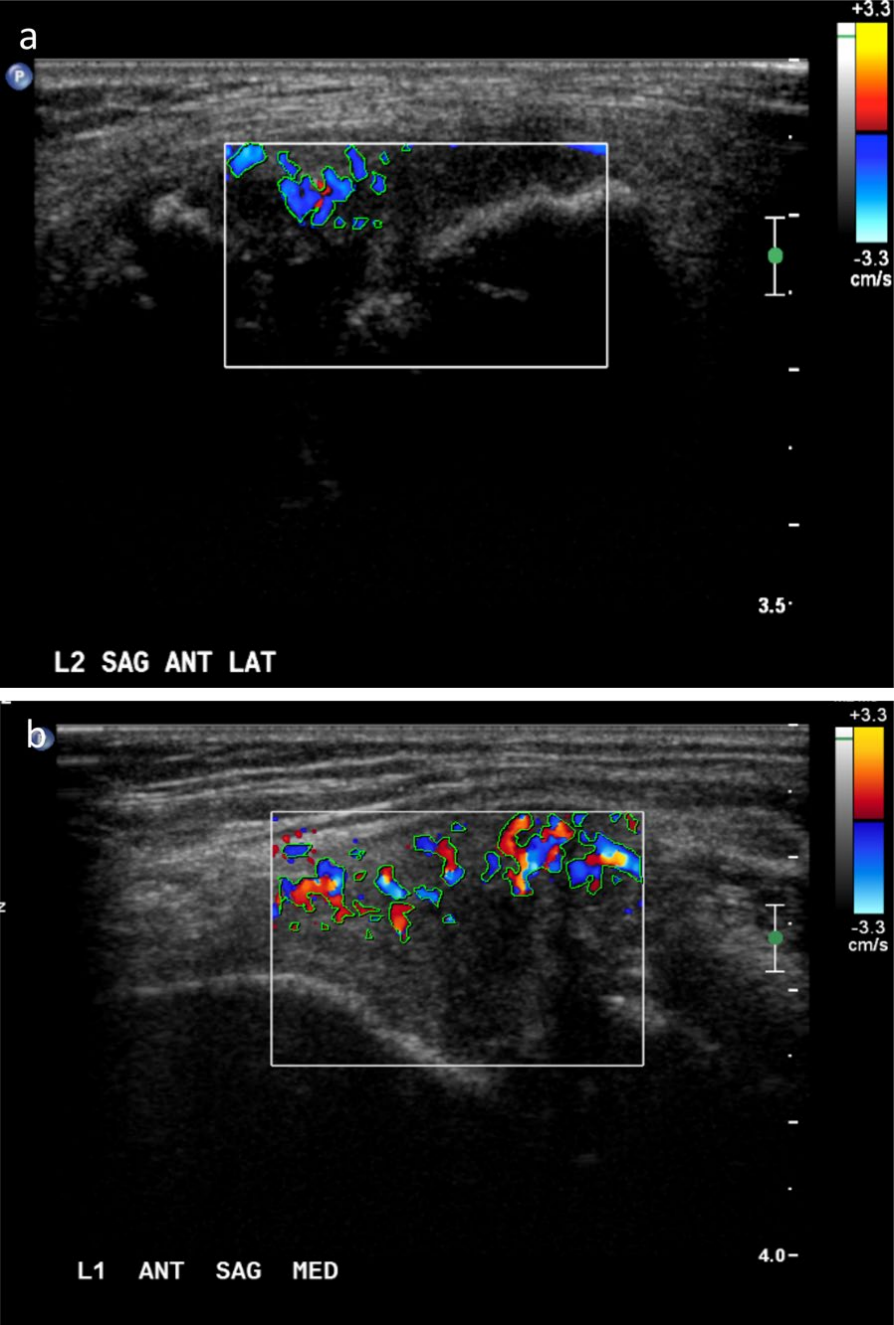
Increased synovial vascularity of joints of patients with hemophilic arthropathy on color Doppler ultrasound. **a** Eight-year old boy with severe hemophilia A and a history of prior bleeding into his right ankle. Mild hypervascularity is seen in his joint on color Doppler ultrasound. Color Doppler sonogram obtained on the sagittal anterior-lateral plane at the L2 level shows more than 4 color pixel dots encompassing less than 50% of the synovium extent within the color Doppler box which represents some degree of synovitis. Score equals 1. The area of color pixels within the color box is 4,257 units^2^. **b** Twelve-year old boy with severe hemophilia A and a history of recurrent bleeding into his left elbow. Extensive hypervascularity is seen in his joint on color Doppler ultrasound. Color Doppler sonogram obtained on the sagittal anterior medial plane at the L1 level shows more than 4 color pixel dots encompassing 50% or more of the synovium extent within the color Doppler box. Score equals 2. The area of color pixels within the color box is 13,605 units^2^

The degree of vascularity of a given joint on color Doppler ultrasound was scored based on the image that showed most severe findings regardless of location in the joint and included the number of color pixels and extent of vascularity seen within the ROI (Figs. 1, 2) according to a semi-quantitative score, modified from Backhaus et al. [24] as follows: ‘normal’ (grade 0), ‘mild to moderate’ (grade 1) and ‘severe’ (grade 2) (Table 1). Two radiologists with 8 and 6 years of experience in ultrasound after training, respectively (reviewer 1, N.Z.; reviewer 2, S.Y.), evaluated each assigned image according to the a priori determined criteria (Table 1). They both completed a tutorial session for calibration of measurements on color Doppler images of joints before formal reading of the study images. Discrepancies in results were resolved by an adjudicator (A.S.D.), a radiologist with over 10 years of experience after training.

**Table 1:** Color Doppler semi-quantitative scoring for assessment of ankles, elbows and knees of patients with blood-induced arthropathies

Grades	Criteria	Score
Normal (grade 0)	Normal: < 4 dots within box	0
Mild to moderate (grade 1)	≥ 4 dots within box and < 50% of ROI filled with color pixels representing hyperemia	1
Severe (grade 2)	≥ 50% of ROI filled with color pixels representing hyperemia	2

Criteria: Based on the number of dots (color pixels) and extent of vascularity at the location with most severe findings and extent of vascularity within the color Doppler box

ROI, region-of-interest

#### MRI

Like measurements attained on gray-scale ultrasound images, synovial thickness was measured on the MRI plane and image that depicted the area with most severe hypertrophic synovium by a single operator (A.F.Z.).

#### Physical examination

The most affected joint underwent physical examination according to the Hemophilia Joint Health Score (HJHS) version 2.1 by an experienced score-trained physiotherapist in each of the two sites in China [28] (Additional file 1: Table 2). The HJHS version 2.0 was used for the site in Canada and scores were converted into the HJHS version 2.1 scale by the study physiotherapist in Toronto.

#### Statistical analysis

Count and area of pixels on color Doppler were reported as mean, median and range/standard deviation (SD) as appropriate.

The inter-reader reliability for the utilization of either a semi-quantitative scoring method (subjective assessment by experienced readers) or a quantitative method of measurement of color pixels (objective assessment) reflecting the maximum degree of vascularity on color Doppler was analyzed by intraclass correlation coefficients (ICCs) for continuous data, Kendall’s coefficient of concordance for ordinal data and kappa coefficients for nominal data (< 0.40, poor agreement; ≥ 0.40 and < 0.60, moderate agreement; ≥ 0.60 and < 0.80, substantial agreement; and ≥ 0.80, excellent agreement) [29]. Associations between the color Doppler synovial scores (subjective assessment), quantitative color Doppler pixel measurements (objective assessment) and quantitative gray-scale US measurements of synovial hypertrophy were compared among themselves and with total Hemophilia Joint Health Score (HJHS) scores: presence/absence of swelling, duration of swelling, flexion/extension loss and pain item of the HJHS score. Pettersson X-ray scores were calculated with Spearman correlation coefficients (*r* values) for continuous data. *r* values < 0.40 indicated poor, ≥ 0.40 and < 0.60 moderate, ≥ 0.60 and < 0.80 substantial and ≥ 0.80 excellent agreement [29]. For assessment of associations between continuous and categorical variables analysis of variance (ANOVA) was utilized.

In some joints in the China and Canada datasets, the synovium thickness could not be reliably assessed by the operators either due to technical limitations or to extremely reduced thickness and represented missing data. Correlations for synovial thickness measured on US in this study took into consideration available values in each dataset. Concerning demographic information of study subjects information on missing data for baseline factor VIII/IX and HJHS scores is available in Additional file 1: Table 2.

*p* values < 0.05 were considered statistically significant. We used SAS® system software package, version 9.4 (SAS Institute Inc. Cary, NC) for all analyses.

## Results

Table 2 and Additional file 1: Table 2 shows demographic and clinical characteristics of study subjects. A total of 63 children and adolescents with ages ranging from 5 to 18 years, median age 12.3 years agreed to participate in the original studies conducted at the three participating hemophilia treatment centers and were imaged by gray and color Doppler ultrasound, 34 in China (site 1, Beijing [*n* = 22]; site 2, Guangzhou [*n* = 12]; and 29 in Canada (site 3, Toronto). Sixty cases of our cohort were boys with severe or moderate hemophilia A [*n* = 52] or B [*n* = 8]. Forty one cases had factor VIII/IX levels of < 1% and 19 cases had factor VIII/IX levels of 1–5%. Three patients had von Willebrand disease (VWD) type 3, a severe form of VWD. There was a good balance of ankles (35.2%, 12 out of 34), knees (32.4%, 11 out of 34) and elbows (32.4%, 11 out of 34) studied in the Chinese cohort but for the Canadian cases the majority of joints studied were ankles (71.4%, 20 out of 28).

**Table 2:** Demographic and clinical characteristics of study subjects

Study site	N	Age (range, median)	Ankles Imaged	Elbows imaged	Knees imaged	Hemophilia type	Baseline FVIII/IX Level* (%)	Pettersson score	HJHS score (range, median)
1	22	7–17, 13	8	7	7	A:N = 17	0:N = 13;	Score 0:N = 1	7–15, 9
							1: N = 4	Scores 1–3:N = 8	
								Scores 4–7:N = 5	
						B:N = 5	0:N = 4	Scores ≥ 8:N = 8	
							1:N = 1		
2	12	8–18, 11.5	4	4	4	A:N = 12	0:N = 5	Score 0:N = 0	3–16, 11.5
							1:N = 7	Scores 1–3:N = 4	
								Scores 4–7:N = 4	
								Scores ≥ 8:N = 4	
3	29	5–17, 13	21	0	8	A:N = 23	0:N = 20	Score 0:N = 14	0–16, 4.5
							1:N = 3	Scores 1–3:N = 4	
						B:N = 3	0:N = 2	Scores 4–7:N = 5	
							1:N = 1	Scores ≥ 8:N = 6	
						vWD type 3:N = 3**			

N, number; FVIII/IX, factors VIII/IX; IU, International Unit; dL, deciliter; HJHS, Hemophilia Joint Health Score

*Baseline FVIII/IX level: 0: < 1 IU/dL (= < 1%, consistent with a diagnosis of severe hemophilia) 1: 1-5 IU/dL ( = 1-5%, consistent with a diagnosis of moderate hemophilia)

**Three patients had vWD

As expected the severity of joint disease at the time of the imaging studies was discordant between the Canadian and Chinese cases: 61.7% (21 out of 34) of the Chinese cases had joints with total HJHS scores of 4 or higher versus 37.9% (11 out of 29) of cases from Canada. This almost certainly reflects the fact most boys < 18 years of age in Canada with moderate/severe hemophilia would have been on some form of long-term prophylaxis for several years whereas few boys with moderate/severe hemophilia in China studied in 2015 would have been on long-term prophylaxis for a significant period of time.

The inter-reader reliability for using different color Doppler methods for assessment of the maximum degree of synovial vascularity, a semi-quantitative scoring method (subjective assessment) and quantitative pixel measurements (objective assessment) was excellent for the semi-quantitative method and moderate/lower range of substantial for the quantitative method regardless of the origin of the data acquisition (Table 3).

**Table 3:** Inter-reader reliability for using different color Doppler methods (subjective, semi-quantitative and objective, quantitative) for assessment of maximal synovial vascularity in joints of patients with blood-induced disorders

Testing domain	US operators (data acquisition)	Reliability [ICC (95% CIs)] for interpretation of data by readers (1, 2)
Semi-quantitative scoring system		
	Canada	1.00 (1.00, 1.00)
	China	0.98 (0.97, 0.98)
Quantitative scoring system		
	China	
	Area of pixels	0.61 (− 0.51, 0.96)
	Count of pixels	0.60 (− 0.52, 0.96)
	Canada	
	Area of pixels	0.67 (0.43–0.82)
	Count of pixels	0.70 (0.48–0.84)

US, ultrasound; ICC, intraclass correlation coefficients; CI, confidence intervals

Concerning the association between the two aforementioned color Doppler analytic methods, semi-quantitative and quantitative  scoring systems, results depended most on the reader who defined the ROIs than on the origin of the data. Whereas highly substantial correlations were noted for both Canada and China data between the two methods when reader 1 defined the ROI, moderate to substantial correlations were noted for Canada and China data, respectively, when ROIs were defined by reader 2 (Table 4).

**Table 4:** Associations between a subjective semi-quantitative color Doppler US scoring method (A), a quantitative color Doppler method for synovial vascularity (B) and a quantitative gray-scale US method for single-layer synovial hypertrophy (C)

Origin of patient’s data	Correlation coefficient *r* (95% CIs)	*p* value	Correlation coefficient *r* (95% CIs)	*p* value
	A vs B methods		B vs C methods	
*A. Canada US operator*				
	**Reader 1**		**Reader 1**	
Canada Data	0.72 (0.47, 0.86)	**< 0.0001**	0.43 (0.08, 0.69)	**0.02**
China Data	0.76 (0.56, 0.87)	**< 0.0001**	0.66 (0.40, 0.82)	**< 0.0001**
	**Reader 2**		**Reader 2**	
Canada Data	0.63 (0.34, 0.81)	**0.0001**	0.44 (0.08, 0.69)	**0.02**
China Data	0.79 (0.62, 0.89)	**< 0.0001**	0.60 (0.34, 0.78)	**< 0.0001**
*B. China US operator*				
	**Reader 1**		**Reader 1**	
Canada Data	0.63 (0.34, 0.81)	**0.0001**	0.44 (0.08, 0.69)	**0.0151**
China Data	0.79 (0.62, 0.89)	**< 0.0001**	0.60 (0.34, 0.78)	**< 0.0001**
	**Reader 2**		**Reader 2**	
Canada Data	0.84 (0.68, 0.92)	**< 0.0001**	0.64 (0.35, 0.81)	**< 0.0001**
China Data	0.85 (0.71, 0.92)	**< 0.0001**	0.61 (0.33, 0.79)	**0.0001**

Bold indicates statistically significant results. *p* values < 0.05 were considered statistically significant

US, ultrasound; CI, confidence intervals

Table 5 shows mean thickness of single layer synovium on gray-scale US and mean count of pixels in the peri-synovial region of joints of patients on color Doppler US. Differences were noted for mean synovial thickness when data from patients of Canada and China were compared, regardless of the country of origin of US operators (*p* < 0.0001 for each comparison) and also for mean count of color Doppler pixels between data from China and Canada patients, regardless of the country of origin of US operators (*p* = 0.003 and *p* = 0.002, for Canada and China US operators, respectively).

**Table 5:** Mean thickness of synovium and mean count of color Doppler pixels in the perisynovial regions of joints of patients from Canada and China centers according to origin of ultrasound operators and patients’ data

Origin of ultrasound operators	Origin of patients’ data	Mean thickness of synovium (mm) - gray-scale US	*p* value (between values according to origin of patients’ data)	Mean count of color Doppler pixels	*p* value (between values according to origin of patients’ data)
Canada	Canada	2.1	*P* < 0.0001	930	*P* = 0.003
Canada	China	4.1		3,633	
China	Canada	2.0	*P* < 0.0001	868	*P* = 0.002
China	China	3.9		3,796	

Single layer synovium was considered in the data analysis

Color Doppler pixel count was used as an outcome of synovial vascularity

US, ultrasound. *p* values < 0.05 were considered statistically significant

Concerning the association between the quantitative degree of vascularity on color Doppler and the extent of synovial hypertrophy on gray-scale ultrasound correlations were consistently at lower limits of moderate for Canada data and upper limits of moderate to substantial for China data for Canadian US operators slightly improving for Canada data when Chinese US operators obtained the data. These correlations were maintained regardless of the reader who performed measurements (Table 4).

Correlations for synovial thickness measured on US between Canadian and Chinese readers were excellent for China data (*r* = 0.93, *p* < 0.0001) and substantial for Canada data (*r* = 0.77, *p* = 0.0001). Nevertheless, correlations for synovial thickness measured on MRI and US were moderate for China data (*r* = 0.64, *p* = 0.001, Canadian US operator and *r* = 0.63, *p* = 0.001, Chinese US operator) and not significant for Canada data (*r* = 0.05, *p* = 0.86, Canadian US operator and *r* = 0.46, *p* = 0.10, China US operator). Note is made of the fact that in 10 out of 34 (29.4%) available cases of the China dataset and in 14 out of 29 (48.3%) cases of the Canada dataset synovium thickness could not be reliably measured by MRI representing missing data.

Table 6 shows associations between the degree of vascularity on color Doppler and other total HJHS scores, presence/absence of swelling, duration of swelling, flexion loss, extension loss, and pain (items of HJHS score)] and Pettersson X-ray scores in the assessment of hemophilic arthropathy.

**Table 6:** Associations between the degree of vascularity on color Doppler and clinical /imaging constructs Hemophilia Joint Health Score (HJHS) of the study joint, Pettersson score of the study joint

US operators (data acquisition)		Correlation coefficient *r* (95% CIs)	*p* value	Correlation coefficient *r* (95% CIs)	*p* value	Correlation coefficient *r* (95% CIs)	*p* value
		Semi-quantitative color Doppler US scoring method (synovial vascularity)		Quantitative color Doppler US pixel measurement (synovial vascularity)		Quantitative gray-scale US measurement (single-layer synovial hypertrophy)	
Canada	Total Pettersson X-rays scores	0.14 (− 0.25, 0.48)	0.49	0.04 (− 0.34, 0.40)	0.85	0.24 (− 0.15, 0.56)	0.22
China	Total Pettersson X-rays scores	0.29 (− 0.06, 0.57)	0.09	0.11 (− 0.25, 0.44)	0.55	0.25 (− 0.10, 0.54)	0.15
Canada	Total HJHS scores	0.21 (− 0.20, 0.55)	0.30	0.31 (− 0.09, 0.62)	0.12	0.53 (0.16–0.75)	**0.005**
China	Total HJHS scores	0.32 (− 0.10, 0.63)	0.12	− 0.01. (− 0.41, 0.42)	0.97	0.16 (− 0.26–0.52)	0.46
Canada	Presence/absence of swelling (item of HJHS score)	0.32 (− 0.06, 0.61)	0.09	0.38 (0.01, 0.65)	**0.04**	0.27 (− 0.12–0.57)	0.16
China	Presence/absence of swelling	− 0.16 (− 0.50, 0.23)	0.41	− 0.20 (− 0.54, 0.21)	0.34	− 0.05 (− 0.41–0.33)	0.81
Canada	Duration of swelling (item of HJHS score)	0.10 (− 0.28, 0.45)	0.61	0.23 (− 0.16, 0.54)	0.24	0.06 (− 0.32–0.41)	0.77
China	Duration of swelling	− 0.01 (− 0.39, 0.36)	0.94	0.12 (− 0.28, 0.48)	0.56	− 0.06 (− 0.42–0.32)	0.77
Canada	Flexion loss (item of HJHS score)	0.24 (− 0.15, 0.55)	0.22	0.16 (− 0.22, 0.50)	0.41	0.07 (− 0.31–0.42)	0.72
China	Flexion loss	0.38 (− 0.00, 0.65)	**0.048**	0.40 (0.00, 0.68)	**0.04**	0.27 (− 0.12–0.58)	0.17
Canada	Extension loss (item of HJHS score)	0.34 (− 0.04, 0.62)	0.07	0.25 (− 0.13, 0.56)	0.18	0.39 (0.01–0.65)	**0.04**
China	Extension loss	0.09 (− 0.30, 0.45)	0.65	− 0.16 (− 0.51, 0.24)	0.43	0.10 (− 0.29–0.45)	0.61
Canada	Pain (item of HJHS score)	0.23 (− 0.16, 0.54)	0.24	0.13 (− 0.25, 0.47)	0.52	0.34 (− 0.04–0.62)	0.07
China	Pain	0.21 (− 0.18, 0.54)	0.28	0.09 (− 0.31, 0.46)	0.68	0.29 (− 0.10–059)	0.14

Bold indicates statistically significant results. *p* values < 0.05 were considered statistically significant

CI, confidence intervals; R, correlation coefficient

## Discussion

The synovial membrane is richly supplied with blood, vessels, lymphatics and nerves. Ultrasonography is non-invasive, inexpensive and readily available with no reported complications. It is ideal for determining the presence, extent and nature of soft tissue changes in joints of persons with hemophilia. It has become a mainstay diagnostic tool in the evaluation of inflamed joints, including reactive synovitis in subjects with inflammatory arthropathy.

In this study, the synovium thickness, as well as the area and count of pixels were objectively measured by gray scale and color Doppler US, respectively. Moreover, synovitis was subjectively graded by a color Doppler US scoring system. Our results showed excellent agreement for inter-reader reliability between subjective and objective assessments of ROIs of joints. Nevertheless, we should note that participation in a tutorial session for calibration of ROIs of synovial inflammation prior to commencement of measurements is key given the subjectivity for selection of ROIs. Previous imaging studies have shown that the utilization of a tutorial session and atlas improves the scoring of inflammatory findings of joints, particularly for non-experienced operators [30]. Note is made that reader 1 had a previous 6-month dedicated US training in the assessment of hemophilic arthropathy.

Our study results indicate an overall poor association between the degree of vascularity on color Doppler and the extent of synovial hypertrophy on gray-scale ultrasound for Canada data and moderate agreement for China data (Table 4). One explanation for this is the fact that for China data categories of absent, mild and severe synovial hypertrophy are available in our dataset whereas for Canada data only absent and mild synovial hypertrophy categories are available. High association means that severe synovial hypertrophy is frequently associated with marked synovial hyperemia, mild synovial hypertrophy with a lower degree of hyperemia and absent synovial hypertrophy with absence of synovial color pixels. Although in both datasets absent synovial hypertrophy is associated with 0 or with a small number of color pixels, the lower number of categories of synovial hypertrophy for the Canada dataset and the more frequent presence of color pixels in cases of absent synovial hypertrophy may justify the poorer associations for Canada data. Further normative data on synovial vascularity according to different levels of physical activity of children is urgently needed for us to better understand the definitions of active and non-active synovium and the relationship between degree of synovial hypertrophy and of reactive synovial hypervascularity. The lower HJHS scores of the study joint for Canada data in relation to China data (Table 2) may infer that joints of Chinese patients had more active synovitis due to more severe joint bleeding than joints of Canadian patients which could account for overall less synovial inflammation and lesser grade of synovial hypertrophy seen on Canada data.

Previous studies on the assessment of synovial abnormalities with ultrasound and MRI showed a good agreement between the two techniques [27]. In our study, the inter-reader reliability for measuring maximum degree of synovial vascularity, showed excellent reliability for semi-quantitative assessment, both in Canada and China data, and moderate/lower range of substantial for quantitative assessment in the data from all three hemophilia treatment centers. This suggests that the proposed semi-quantitative color Doppler holds more promise for use in clinical practice, regardless of the severity of synovitis. Furthermore, in this study unenhanced MRI examinations were not useful as comparators for synovium thickness in relation to gray-scale US given the impossibility of reliable measurements to be obtained without contrast enhancement and the presence of susceptibility artifacts from hemosiderin deposition on gradient-recalled echo images which obscured fine detail of the synovium. These limitations of MRI yielded missing data for synovium thickness on MRI. US has advantages over MRI. One of these advantages is its ability of differentiating synovium hypertrophy and hemosiderin deposition, which may not possible with some sequences of MRI in cases of severe deposition of extracellular hemosiderin due to susceptibility artifacts, for example, on gradient-echo images. Although contrast-enhanced MRI could be of value in joints of patients with bleeding disorders with no or minimal hemosiderin deposition it is of reduced value in joints with substantial hemosiderin deposition because the synovium is obscured by dark signal from hemosiderin on post-contrast MRI sequences [31, 32].

Also, the smaller field-of-view of US in comparison to MRI allowed for more detailed evaluation of synovium by US than by MRI. As a result, the assessment of associations of synovium thickness between MRI and gray-scale US was not robust. Of interest, using dynamic contrast-enhanced MRI in their study Acharya et al. [9] reported strong correlations between measurements of synovial thickness (*r* = 0.70, *p* < 0.0001) and synovial vascularity (*r* = 0.73, *p* < 0.0001) on power Doppler US and those of MRI. They found a sensitivity of 100% and a specificity of 94% for a cutoff of power Doppler US intensity of 1.3 decibels in 17 joints of children and adults with hemophilia A/B and other inherited bleeding disorders with/without a history of hemarthrosis and ages ranging from 6 to 60 years.

A limitation of the study was the unavailability of accurate information about timing of the bleeding history given the retrospective nature of this study. Synovial vascularity scores used in this study were based on the assessment of reactive synovitis which would be indirectly related to recent bleeding compared with previous bleeding history. Previous studies have addressed the lack of accuracy of patients’ reports on occurrence of joint bleeds [33], which favours the use of the Hemophilia Joint Health Score (HJHS) as a more objective tool for assessment of the joint health of patients with hemophilia. The HJHS scores was the one of the diagnostic outcome measures used in our study for this reason.

The remaining correlations between subjective assessment of synovitis (scoring) and clinical (HJHS total score and score of items) and radiological (Pettersson X-ray) scores were all poor. These results raise the importance of complementary utilization of physical examination and US as part of clinical practice as previously reported [34]. Note should be made that the synovial hypertrophy scores used in this study were based on the location with maximal synovial thickness in the joint, whereas many of the HJHS items assessed represented either different items (e.g. swelling on HJHS vs joint effusion/hemarthrosis ± synovial hypertrophy on gray-scale US) or conjoint soft tissue and osteochondral joint findings (e.g. extension and flexion loss, pain vs multiple items on gray-scale US). Furthermore, synovial vascularity scores used in this study were based on the assessment of reactive synovitis which would be indirectly related to synovial hypertrophy.

The lack of a reference standard for measurements of synovial vascularity for which contrast would have to have been administered to patients is a limitation of this study. For this reason, in addition to superposition of hemosiderin deposition to synovium in many examinations, it was difficult to measure the synovial thickening on corresponding MRI examinations. Nevertheless, the use of contrast-enhanced MRI for assessment of joints of patients with hemophilia is not the standard practice in many centers [31] and was not part of this study. Second, the imaging data from the cohorts in Canada and China were obtained in different timeframes which could have posed differences in quality of imaging between the Canadian and Chinese centers. Nevertheless, the types of probes, US scanner manufacturer, protocol and technical parameters used were similar which made comparable the datasets Third, the HJHS scores used in the China cohorts (sites 1 and 2) and Canada (site 3) cohorts were slightly different (versions 2.1 and 2.0, respectively). Also, elbow joints were only assessed in the China cohorts. Finally, spectrum bias was noted in the cohorts considering that overall the disease burden was significantly greater in the China cases and the joints studied in the Canadian cases were predominantly ankles.

The excellent inter-reader reliability noted for the use of different color Doppler methods (subjective vs objective) for assessment of the maximum degree of synovial vascularity (Table 3) supports the utilization of synovitis scores for assessment of blood-induced arthropathies in clinical practice.

Our study shows moderate to substantial associations between quantitative degree of synovial vascularity on color Doppler and extent of synovial hypertrophy on gray-scale US on data from both Canada and China, but most notably for China data (Table 4). These results raise the possibility that reactive synovitis can help in determining timing of prior joint bleeds (clinical/subclinical) if color Doppler measurements are obtained close in time to the date of the joint bleed. This hypothesis should be investigated in prospective studies with objective criteria being defined upfront.

The overall non-significant correlations observed between measurements of degree of synovial vascularity by color Doppler US and HJHS scores (Table 6) point out to the need for complementary use of US and physical examination in clinical practice. This is due to the fact that different domains (individual vs conjoint joint findings) require evaluation by different diagnostic tests.

Although demographic results of this study have shown that overall the average gray-scale US thickness of single-layer synovium was smaller for Canada data than for China data, results of Table 4 could well represent differences in the available data in the Canadian and Chinese cohorts. In the Canadian cohort, 8 knees and 21 ankles were examined whereas in the Chinese cohort, 11 knees, 12 ankles and 11 elbows were examined with minimal to mild disease noted in the Canadian cohort in contrast to the degree of severity of arthropathy in the Chinese cohort) which resulted in statistically significant *r* value levels that most likely are not clinically meaningful (*r* < 0.50).

## Conclusion

Color Doppler US is a valuable scoring method for evaluating reactive synovitis in joints of subjects with inherited bleeding disorders and holds potential for assessing post-bleed reactive synovitis in a generalizable worldwide way once further information on its association with timing of the joint bleed becomes available in the literature.

## Supplementary Information

**Additional file 1.Supplementary Tables. Table 1.** MRI protocols used in the three participating centers. **Table 2.** Demographic and clinical characteristics of study subjects.

## Data Availability

All data generated or analyzed during this study are included in this published article and its supplementary information files.

## Abbreviations

ANOVA: Analysis of Variance
HJHS: Hemophilia Joint Health Score
ICCs: Intraclass Correlation Coefficients
JIA: Juvenile Idiopathic Arthritis
MHz: MegaHertz
MRI: Magnetic Resonance Imaging
PACS: Picture Archive Communications Systems
ROIs: Regions of Interest
SD: Standard Deviation
US: Ultrasound
VWD: Von Willebrand disease
