# Supplementary material for: A semiquantitative color Doppler ultrasound scoring system for evaluation of synovitis in joints of patients with blood-induced arthropathy
Source: Insights Imaging. 2021 Sep 25;12:132. doi: 10.1186/s13244-021-01043-0 (PMC8464640; doi:10.1186/s13244-021-01043-0)
Supplement: Supplementary file 1 — Additional file 1.Supplementary Tables. Table 1. MRI protocols used in the three participating centers. Table 2. Demographic and clinical characteristics of study subjects. [file 13244_2021_1043_MOESM1_ESM.docx]

**Supplementary Tables**

**Table 1. MRI protocols used in the three participating centres.**

**Site 1**

| Sequences | Planes | TR（ms） | TE  （ms） | FOV  (mm) | Thickness  (mm) | Interval  （mm） | Matrix | NEX | Scanning time (min) |
| --- | --- | --- | --- | --- | --- | --- | --- | --- | --- |
| T2_FFE | Sagittal | 422 | 12 | 160 | 3 | 0.5 | 304*253 | 2 | 2:54 |
| T2WI_FFE | Coronal | 422 | 12 | 160 | 3 | 0.3 | 382*262 | 1 | 3:45 |
| T2WI_FFE | Axial | 422 | 12 | 160 | 3 | 0.3 | 382*262 | 1 | 3:45 |
| T2WI_SPAIR | Sagittal | 4000 | 80 | 160 | 3 | 0.3 | 248*182 | 2 | 4:00 |
| PDW_TSE | Sagittal | 4817 | 30 | 160 | 3 | 0.5 | 360*276 | 2 | 3”08 |
| T1W_TSE | Coronal | 500 | 15 | 160 | 3 | 0.5 | 292*272 | 1 | 2”06 |

**Site 2**

| Sequences | Planes | TR（ms） | TE（ms） | FOV | Thickness（mm） | Interval（mm） | Matrix | NEX | Scanning time |
| --- | --- | --- | --- | --- | --- | --- | --- | --- | --- |
| T2WI_FFE | Sagittal | 422 | 12 | 160 | 3 | 0.3 | 382*262 | 1 | 3:45 |
| T2WI_FFE | Coronal | 422 | 12 | 160 | 3 | 0.3 | 382*262 | 1 | 3:45 |
| T2WI_FFE | Axial | 422 | 12 | 160 | 3 | 0.3 | 382*262 | 1 | 3:45 |
| T2WI_SPAIR | Sagittal | 4000 | 60 | 160 | 3 | 0.3 | 248*182 | 2 | 4:00 |
| PDWI | Sagittal | 4259 | 30 | 160 | 3 | 0.5 | 364*263 | 2 | 2:55 |
| T1WI | Coronal | 500 | 20 | 160 | 3 | 0.5 | 380*299 | 2 | 2:05 |
| 3D_WATS | Sagittal | 20 | 6.7 | 160 | 3 | 1.5 | 572*571 | 1 | 8:56 |
|  |  |  |  |  |  |  |  |  |  |

**Site 3**

| Sequences | Planes | TR（ms） | TE  （ms） | FOV  (mm) | Thickness  (mm) | Interval  （mm） | Matrix | NEX | Scanning time |
| --- | --- | --- | --- | --- | --- | --- | --- | --- | --- |
| 3D gradient-echo | Sagittal | 400 | 15 | 130 | 4 | 0.4 | 256*192 | 2 | 2:16 |
| 3D gradient-echo | Coronal | 467 | 15 | 130 | 4 | 0.4 | 256*192 | 2 | 2:26 |
| 3D gradient-echo  3D | Axial | 467 | 15 | 130 | 4 | 0.4 | 256*192 | 2 | 2:32 |
| T2W_SE | Sagittal | 3500 | 92 | 140 | 3 | 3.3 | 256*224 | 2 | 3:57 |
| PDW_TSE | Sagittal | 3000 | 42 | 130 | 3 | 3.3 | 256*256 | 2 | 3:22 |
| T1W_TSE | Coronal | 516 | 12 | 140 | 3 | 3.3 | 256*192 | 2 | 2:38 |
| _SPGR FS (High Res)_ | Sagittal | 21.6 | 5.5 | 160 | 1.5 | 0 | 512 x 512 | 1 | 8:48 |
|  |  |  |  |  |  |  |  |  |  |

**Abbreviations:** TR, repetition time; TE, echo time; ms, miliseconds; mm, millimeters; FOV, field-of-view; NEX, number of excitations; FA = flip angle, W, weighted; SE, spin-echo; TSE, turbo spin-echo; SPGR, spoiled gradient recalled; high res, high spatial resolution.

Note: The protocol described in reference 24 relates to the protocol used in site 2 which differs from the aforementioned protocol used in site 1 of that study.

**Table 2. Demographic and clinical characteristics of study subjects.**

| **Study site** | **Age** | **Joint Imaged** | **Haemophilia type** | **Baseline FVIII/IX** | **HJHS score** |
| --- | --- | --- | --- | --- | --- |
| Site 1 | 8 | R ankle | A | 4 | 9 |
| Site 1 | 16 | R knee | A | 4 | 15 |
| Site 1 | 14 | L elbow | A | 1 | 10 |
| Site 1 | 7 | L elbow | A | 0 | 7 |
| Site 1 | 12 | L elbow | A | missing | 11 |
| Site 1 | 17 | R elbow | B | missing | 8 |
| Site 1 | 15 | L elbow | A | missing | 13 |
| Site 1 | 14 | R knee | A | missing | 9 |
| Site 1 | 12 | L ankle | A | missing | missing |
| Site 1 | 10 | R ankle | A | 1 | missing |
| Site 1 | 12 | L elbow | A | 1 | missing |
| Site 1 | 17 | L elbow | B | 0 | 14 |
| Site 1 | 13 | R knee | A | 1 | missing |
| Site 1 | 13 | L knee | A | 1 | 8 |
| Site 1 | 17 | R ankle | A | 1 | 7 |
| Site 1 | 14 | R ankle | A | 2 | 7 |
| Site 1 | 8 | R knee | B | 1 | 12 |
| Site 1 | 8 | R ankle | B | 0 | 8 |
| Site 1 | 9 | R knee | B | 2 | 8 |
| Site 1 | 7 | L ankle | B | 1 | missing |
| Site 1 | 16 | L ankle | A | 0 | 9 |
| Site 1 | 17 | L knee | A | 0 | 13 |
| Site 2 | 13 | R ankle | A | 1 | 11 |
| Site 2 | 15 | L elbow | A | 1 | 13 |
| Site 2 | 11 | L elbow | A | 1 | 3 |
| Site 2 | 15 | R knee | A | 1.1 | 12 |
| Site 2 | 18 | R ankle | A | 1 | 9 |
| Site 2 | 9 | R knee | A | 1 | 12 |
| Site 2 | 8 | L elbow | A | 2.6 | 10 |
| Site 2 | 8 | L ankle | A | 2 | 10 |
| Site 2 | 15 | R knee | A | 0.8 | 14 |
| Site 2 | 12 | L elbow | A | 0.8 | 16 |
| Site 2 | 8 | R knee | A | 1 | 12 |
| Site 2 | 10 | R ankle | A | 0.9 | 6 |
| Site 3 | 13 | R ankle | A | 0 | 13 |
| Site 3 | 15 | R knee | A | 0 | 7 |
| Site 3 | 16 | L knee | A | 0 | 6 |
| Site 3 | 17 | R knee | A | 0 | 8 |
| Site 3 | 15 | L knee | A | 0 | 12 |
| Site 3 | 11 | L ankle | A | 0 | 6 |
| Site 3 | 10 | L ankle | A | 0 | 7 |
| Site 3 | 12 | L ankle | T3vWD | 2 | 6 |
| Site 3 | 15 | R ankle | A | 1 | 7 |
| Site 3 | 14 | L knee | A | 0 | 3 |
| Site 3 | 17 | L ankle | A | 0 | 3 |
| Site 3 | 8 | R ankle | A | 0 | 0 |
| Site 3 | 13 | L ankle | B | 0 | 4 |
| Site 3 | 15 | R ankle | A | 0 | 6 |
| Site 3 | 15 | R ankle | A | 0 | 5 |
| Site 3 | 6 | L ankle | A | 0 | 6 |
| Site 3 | missing | missing | missing | missing |  |
| Site 3 | 8 | R knee | A | 0 | 3 |
| Site 3 | 9 | R knee | A | 0 | 1 |
| Site 3 | 6 | L ankle | A | 0 | 2 |
| Site 3 | 17 | R ankle | vWD type 3 | 1 | 16 |
| Site 3 | 8 | R ankle | A | 0 | 1 |
| Site 3 | 17 | R knee | A | 0 | 3 |
| Site 3 | 13 | L ankle | B | 0 | 4 |
| Site 3 | 17 | L ankle | A | 0 | 3 |
| Site 3 | 5 | R ankle | vWD type 3 | 1 | 4 |
| Site 3 | 13 | R ankle | A | 0 | 3 |
| Site 3 | 13 | L ankle | B | 2 | 7 |
| Site 3 | 9 | L ankle | A | 1 | 4 |

**Note:** --R: right; L: left; A: factor VIII; B: actor IX.

--Site1: *BLINDED COUNTRY 2*; site 2: *BLINDED COUNTRY 2*; site 3: *BLINDED COUNTRY 1*

**Abbreviations:** N, number; FVIII/IX, factors VIII/IX; HJHS, Hemophilia Joint Health Score.
